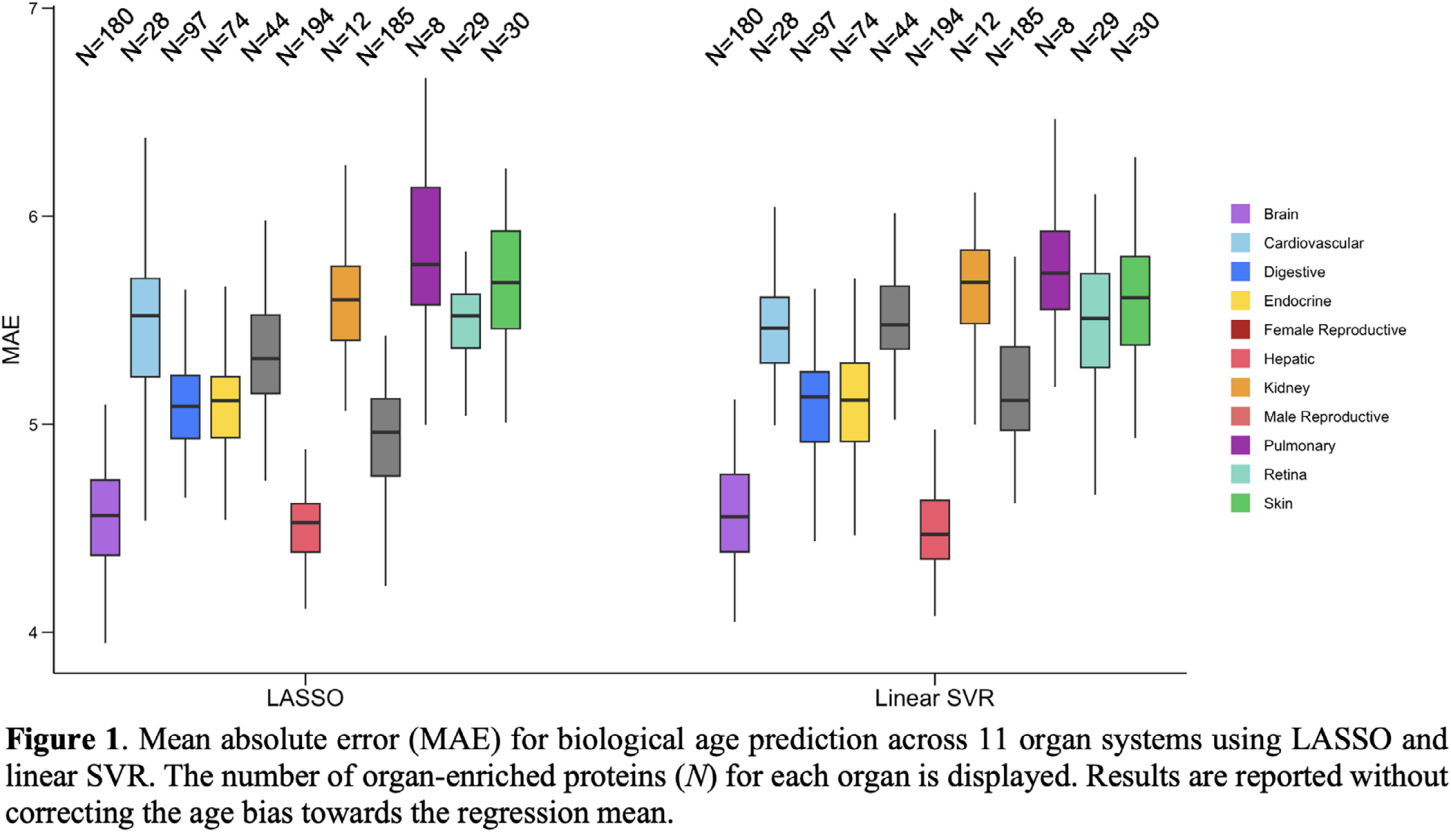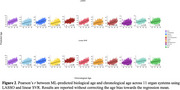# ProtBAG: eleven organ‐specific proteome‐based biological age using CSF proteomics

**DOI:** 10.1002/alz70862_110193

**Published:** 2025-12-23

**Authors:** Sarah Ko, Hui Cao, Mehrshad Saadatinia, Gao Wang, Elisa Konofagou, Donald Edmondson, Wenjia Bai, Arthur W. Toga, Christiane Reitz, Ye Ella Tian, Andrew Zalesky, Christos Davatzikos, Junhao Wen

**Affiliations:** ^1^ Laboratory of AI and Biomedical Science (LABS), Columbia University, New York, NY USA; ^2^ LABS, Columbia University, New York, NY USA; ^3^ Columbia University, New York, NY USA; ^4^ ICL, London, London UK; ^5^ Laboratory of Neuro Imaging, Stevens Neuroimaging and Informatics Institute, Keck School of Medicine, University of Southern California, Los Angeles, CA USA; ^6^ Columbia University, The Taub Institute for Research on Alzheimer’s Disease and the Aging Brain, The Gertrude H. Sergievsky Center and Departments of Neurology and Epidemiology, New York, NY USA; ^7^ Melbourne Neuropsychiatry Centre, Department of Psychiatry, Melbourne Medical School, The University of Melbourne, Melbourne, VIC Australia; ^8^ Center for AI and Data Science for Integrated Diagnostics, University of Pennsylvania, Philadelphia, PA USA; ^9^ New York Genome Center, New York, NY USA

## Abstract

**Background:**

Recent research^1,2^ has generated increasing interest in modeling human aging and disease within a multi‐organ framework. Plasma proteomics^3^ has emerged as a widely used approach for predicting individual chronological age, resulting in the proteome‐based biological age gap (ProtBAG). Here, we used CSF proteomics from the ADNI study to derive 11 organ‐specific ProtBAGs using 2 machine learning (ML) methods.

**Method:**

CSF proteomics was generated using the SomaScan 7k platform in ADNI, which included 7,008 protein levels from 736 participants (mean age: 73.3 ± 7.4 years; 57% women). Missing proteomics values were imputed using AutoComplete^4^. Organ‐enriched proteins for 11 organ systems were determined by at least four‐fold higher mRNA levels in the tissue of interest than in other organ tissues. These organ‐enriched proteins were then fit to a linear support vector regression (SVR) and LASSO regression model. Nested random holdout cross‐validation (50 repetitions) was implemented; mean absolute error (MAE) and Pearson’s *r* were used to evaluate model performance.

**Result:**

For proteomics imputation, we chose the imputed results with the copy‐mask amount of 0.3 (2% missing rate in data), which led to the best model performance (*r* ^2^=0.54). Overall, LASSO and Linear SVR achieved comparable MAE values with only slight differences between the two models. The brain and hepatic showed the lowest MAE values (Linear SVR: 4.56 and 4.52 for the brain and hepatic ProtBAGs; LASSO 4.55 and 4.52 for the brain and hepatic ProtBAGs) (Figure 1). Across the 11 organ systems, MAE values from our analyses, ranging from 4.5 to 6, were in line with previous literature using brain imaging^2^. In addition, the brain and hepatic showed the highest Pearson’s *r* values (Linear SVR: 0.62 and 0.64 for the brain and hepatic ProtBAGs; LASSO 0.63 and 0.65 for the brain and hepatic ProtBAGs) (Figure 2). Other organ systems, such as the endocrine, female reproductive system, and male reproductive system, showed relatively inferior model performance.

**Conclusion:**

This study leverages CSF proteomics data from ADNI to accurately develop 11 organ‐specific ProtBAGs, enriching the organ aging clock framework established in previous literature using plasma proteomics. Future research will investigate the relationship between these ProtBAGs, cognition, and AD progression.